# Changes in prevalence of mental disorders among internally displaced persons in central Sudan: a 1-year follow-up study

**DOI:** 10.1017/gmh.2020.16

**Published:** 2020-09-07

**Authors:** Zienat Sanhori, Edvard Hauff, Arne H. Eide, Ibrahimu Mdala, Abdullah Abdelrahman, Cathrine Brunborg, Lars Lien

**Affiliations:** 1 Federal Ministry of Health, Khartoum, Sudan, P.O. Box 303 Khartoum, Sudan; 2 Institute of Clinical Medicine, Division of Mental Health and Addiction, University of Oslo, Oslo, Norway; 3 Unit for Education and Professional Development, Oslo University Hospital, Norway; 4 SINTEF Digital, Health Research. P.O.Box 124, 0314 Blindern, Oslo, Norway; 5 Department of General Practice, Institute of Health and Society, University of Oslo, Norway, P.O. Box 1130, 0318 Blindern, Norway; 6 Department of Psychiatry, University of Khartoum. P.O. Box 102, Elgasr Street, Khartoum 11115, Sudan; 7 Oslo Centre for Biostatistics and Epidemiology, Research Support Services, Oslo University, Oslo, Norway; 8 Department of Health and Social Sciences, Innlandet University College, P.O. Box 400, 2418 Elverum, Norway; 9 Innlandet Hospital Trust, PO Box 104, 2381 Brumunddal, Norway

**Keywords:** IDPs, mental disorders, Sudan, mental distress

## Abstract

**Background:**

Sudan has one of the largest numbers of internally displaced persons (IDPs) in the world, estimated at five million. The main cause of displacement was the civil war. Attention to the health and in particular the mental health of IDPs has been lacking. That includes limited population longitudinal data describing the “natural” fluctuations of mental morbidity among these groups. The aim of this study is to investigate the level and stability of mental disorders among IDPs over a 1-year period.

**Method:**

In this 1-year follow-up of IDPs in two settlement areas in central Sudan, 1549 persons 18 years or older were interviewed twice using the MINI International Neuropsychiatric Interview. Trained psychologists collected the data in a random household survey in the selected IDP areas.

**Results:**

We found overall high stability among those having and those free of mental disorders in this 1-year follow-up study. There were, however, discernible and statistically significant increases in overall new cases of mental disorders from T1 to T2 as major depression increased by 1.4%, generalized anxiety by 2.8% and social phobia by 1.4%.

**Conclusion:**

The study revealed continued high levels and increases of mental disorders over time, although with a pattern of substantial persistence among those initially ill and limited recovery. This might be due to a complex set of factors such as unavailability of mental health services, poverty, low educational level and social exclusion.

## Background

The number of internally displaced persons (IDPs) globally has steadily increased from around 17 million in 1998 to 45.7 million in 2019 (IDMC, 2020). Most IDPs live in low-income, conflict-affected countries. Forced IDPs are not protected by international refugee laws (Thomas and Thomas, 2004; Siriwardhana and Stewart, 2013), often preventing them from receiving health services, increasing their vulnerability (Thomas and Thomas, 2004). The health and in particular mental health, of IDPs have not been well addressed (Thapa and Hauff, 2005).

Sudan has one of the largest populations of IDPs in the world (Salah *et al*., 2013). The most frequent cause of displacement is civil war which started with the colonial independence in 1956. The second civil war displaced around four million people and new conflicts have developed elsewhere in Sudan. The estimated number of IDPs in Sudan is between 4.5 and 5.2 million.

Studies indicate that IDPs differ from the rest of the population with regard to the type and severity of mental illnesses (Neuner *et al*., 2004; Porter and Haslam, 2005). A study from one of the IDP camps in Darfur reported that 31% were suffering from major depression, 61% from symptoms of depression, and 5% from suicidal ideation. Moreover, there were 21 suicides in a population of 9000 within 1 year, which is ten times the suicide rate of the general population (Kim *et al*., 2007).

Clinical trials have demonstrated that mental disorders can be managed effectively through affordable and locally available interventions in community and primary care settings (Patel and Kleinman, 2003; Patel *et al*., 2007). In order to establish and scale up such mental health services in IDP communities, there is a need for more knowledge about the extent and course of mental health problems. The aim of this study is to investigate changes in mental disorders among IDPs during a 1-year follow up.

## Methods

### Design and setting

A 1-year follow-up community-based study was carried out in 2011 among adult IDPs, in collaboration with the Federal Ministry of Health. The sampling frame was all IDPs settlement areas in Central Sudan, of which two IDP settlement areas were randomly selected among IDP settlement areas listed in the official registry: Mayo, an urban area in the outskirts of the Sudanese capital Khartoum; and Mobi, a rural area in Gezira in Central Sudan (Salah *et al*., 2013). A limited mhGAP training of health workers was implemented in both areas between baseline and follow-up (^†^^1^). The populations of the two study areas migrated from different parts of Sudan from the outbreak of the civil war in 1983–2009 and during the 1983–1985 droughts and famines affecting Darfur and Kordofan. There is a health centre and a rural hospital near Mayo and a health centre in Mobi.

### Participants

The participants were adults from different ethnic groups, speaking different languages, but Arabic was the most common language (Kim *et al*., 2007). A random household sampling of subjects aged 18 and older was carried out. Written consent was obtained before participation in the study. We excluded persons who did not speak Arabic or declined to give informed consent. During October 2011 (Phase1), a total sample of 1876 subjects was included. If anyone was absent at the visit, the house was revisited at an arranged time. The response rate was 97% in phase 1 and 1549 of these subjects were re-interviewed after 1 year (phase 2), for a follow-up response rate of 82.6%.

### Data collection

The data were collected by 20 clinical psychologists who were equally divided into two groups (for Geziraand Khartoum), with equal gender representation. The research team underwent a 1-week training in research interview techniques. As part of the training, a pilot study was carried out among 100 university students in Khartoum city that resulted in suggestions regarding the clarity of language and logistics required for the study. Community guides facilitated community awareness and response. Each data collection team consisted of four members – two interviewers and two community guides or helpers. They randomly directed the household selection using the community health centre as the starting point. Clear marks were made after every household had been interviewed to enable the same households to be accurately identified in the second phase. All household members above 18 years of age were interviewed.

## Measurements

The same instruments including socio-demographic variables (age, gender, education, marital status, occupation, and annual family income) were used at both phases. The International Neuropsychiatric Interview (MINI) is a short structured diagnostic interview, developed jointly by researchers and clinicians for DSM-IV and ICD-10 psychiatric disorders (Hergueta *et al*., 1998). With an administration time of approximately 21 minutes, it was designed to meet the need for a short but accurate structured psychiatric interview for multicentre clinical trials and epidemiology studies (Lecrubier *et al*., 1997). MINI has been translated and used in many languages, including Arabic and it has been applied in various cultures and settings (Eytan *et al*., 2007). Validity and reliability studies have compared MINI to the Structured Clinical Interview for DSM-IV disorders and the Composite International Diagnostic Interview (Hergueta *et al*., 1998). MINI consists of standardized, structured, closed-ended questions. For each disorder, one or two screening questions rule out the diagnosis when answered negatively (Lecrubier *et al*., 1997). No internal reliability score is available for MINI since it applies categories and not a scale.

### Statistical analysis

Descriptive statistics were applied. Socio-demographic characteristics at baseline and prevalence of mental disorders were presented as frequencies (*n*) and proportions (%), or as medians with inter quartile range (IQR), defined by upper quartile (Q3) and lower quartile (Q1). The Mann–Whitney U test was used to compare differences in continuous variables between Khartoum and Gezira. Associations between categorical variables at baseline were established from Chi-square tests. The McNemar test was used to determine whether the prevalence of mental disorders at phase1 had increased or decreased by phase2. All analyses were performed using IBM SPSS Statistics 24 (BM Corp., Armonk, NY).

## Results

A total of 1549 subjects were included in the study. Socio-demographic characteristics of respondents from both study areas, Khartoum (*n* = 849, 54.8%) and Geziera (*n* = 700, 45.2%), are showed in Table 1. Median age of the total sample was 28 years (range: 18–75) and the majority were women (58.0%). Most of the respondents were married (68.6%), were originally from western Sudan (50.7%) and more than half (64.1%) were unemployed. Economic and educational status varied, 46.7% had an income of less than 200 Sudanese Dinar (s.d.) per month and 18.9% had no formal education. Migration due to war was reported by 90%. Participants living in Khartoum were more often married, unemployed, had higher household income, and a higher amount of migration due to war compared to participants living in Gezira (Table 1).

**Table 1.** Socio-demographic characteristics of the 1549 IDPs at baseline by place of residence in Sudan

Socio-demographic	Khartoum (*n* = 849)	Gezira (*n* = 700)	Total (*N* = 1549)	*P* value
Age in years, median (Q1, Q3)	28.0 (22.0, 37.0)	30 (22.0, 37.0)	28.0 (22.0, 37.0)	0.31
Family size, median (Q1, Q3)	6.0 (4.0, 8.0)	6.0 (4.0, 8.0)	6.0 (4.0, 8.0)	0.78
Length of stay, median (Q1, Q3)	18.0 (10.0, 22.0)	18.0 (10.0, 24.0)	17.0 (10.0, 23.0)	0.92
Gender, *n* (%)				
Male	339 (39.9)	311 (44.4)	650 (42.0)	0.07
Female	510 (60.1)	389 (55.6)	899 (58.0)	
Marital status, *n* (%)				
Single	241 (28.4)	245 (35.0)	486 (31.4)	0.01
Married	608 (71.6)	455 (65.0)	1063 (68.6)	
Level of education, *n* (%)				
Illiterate	139 (16.4)	153 (21.9)	292 (18.9)	0.01
Khalwa	220 (25.9)	127 (18.1)	347 (22.4)	0.01
Elementary	317 (37.3)	304 (43.4)	621 (40.1)	0.01
Secondary and above	173 (20.4)	116 (16.6)	289 (18.7)	0.06
Employment status, *n* (%)				
Unemployed	569 (67.0)	424 (60.6)	993 (64.1)	0.01
Temporary	173 (20.4)	175 (25.0)	348 (22.5)	0.03
Permanent	107 (12.6)	101 (14.4)	208 (13.4)	0.30
Household income, *n* (%)				
Less than 200 s.d./per month	376 (44.3)	348 (49.7)	724 (46.7)	0.03
More than 200 s.d./per month	473 (55.7)	352 (50.3)	825 (53.3)	
Place of origin, *n* (%)				
North (Northern States, River Nile)	17 (2.0)	15 (2.1)	32 (2.1)	0.89
South Sudan	72 (8.5)	111 (15.9)	183 (11.8)	0.01
East (Red Sea, Kassala, Gadarif)	59 (6.9)	44 (6.3)	103 (6.6)	0.64
West (Kordofan, Darfur)	456 (53.7)	330 (47.1)	786 (50.7)	0.01
Middle (Khartoum, Gaziera, Sennar, Damazeen)	245 (28.9)	200 (28.6)	445 (28.7)	0.90
Reason for forced migration				
War	816 (96.1)	635 (90.7)	1451 (93.7)	< 0.01
Famine and drought	33 (3,9)	65 (9.3)	98 (6.3)	

For Peer Review.

In phase 1, major depression was the most prevalent disorder reported by 24.6%, followed by generalized anxiety disorder (23.2%), social phobia (14.5%), and PTSD (12.2%). At phase2, the prevalence of major depression and GAD increased to 26%, whereas the prevalence of social phobia and

PTSD increased to 15.9% and 13.0% respectively (Table 2). Psychotic disorders (1%) and alcohol abuse (0.3%) were less prevalent at phase 1 (data not shown in table).

**Table 2.** Prevalence and change in prevalence in the four most common mental disorders (*n* = 1549)

Mental disorder	With disease P 1		With disease P 2		Stable with disease P1–P2		Recovered cases		New cases		*P* value
	*n*	%	*n*	%	*n*	%	*n*	%	*N*	%	
Major depression	381	24.6	402	26.0	213	13.8	15	0.10	36	2.3	<0.01
Social phobia	225	14.5	246	15.9	213	13.8	12	0.80	33	2.1	<0.01
PTSD	189	12.2	201	13.0	182	11.7	7	0.50	19	1.2	0.03
Generalized anxiety disorder	360	23.2	403	26.0	346	22.3	14	0.90	57	3.7	<0.01
Any mental disorder^a^	685	44.2	745	48.1	674	43.5	11	0.70	71	4.6	<0.01

a Participants having at least one mental disorder.

The prevalence of any mental disorder assessed in phase 2 (48.1%) was significantly higher than in phase 1 (44.2%) (*p* < 0.01). But overall, the distribution of types of, and the specific individuals with, disorders remained remarkably stable over time. A total of 674 (43.5%) subjects were diagnosed with at least one mental disorder at both phases,793 (51.2%) subjects were not diagnosed with any mental disorders at either phase. Eleven subjects (0.7%) had recovered and 71 (4.6%) new cases with at least one mental disorder diagnosis were identified at phase 2. Since several subjects had more than one diagnosis, the total number of new diagnosis was145 at phase2. General anxiety disorder had the highest number of new diagnosis (*n* = 57, 3.7%), while the strongest reduction in diagnosis was observed for major depression, and was quite small (*n* = 15, 0.1%)at phase 2 (Table 2).

Table 3 shows the distribution of combinations of mental disorders between phase 1 and 2. A total of 685 subjects had any diagnosis, with 352 (22.7%) subjects having only one diagnosis, 216 (13.9%) subjects had two, 97 (6.2%) had three, and 20 (1.3%) had four diagnoses at phase 1. The number of persons with any diagnoses increased in phase 2 to 745 (as above, reflecting only 11 recoveries and 71 new cases); 386 (24.9%) subjects had only one, 232 (14.9%) subjects had two, 106 (6.8%) had three, and 21 (1.4%) had all four diagnoses (*p* < 0.001). Of the 85 subjects diagnosed with only major depression at phase1, 76 of the same subjects still had major depression at phase 2, nine cases were recovered, 15 had one additional diagnosis, and two had two new diagnoses ending up with 93 subjects diagnosed with major depression in phase 2 (*p* = 0.169), with or without an additional diagnosis. Having both major depression and general anxiety disorder was the most frequent combination of disorders and this frequency was stable between phase1 and phase 2, with 130 participants at phase1 and 132 at phase 2 (*p* = 0.856). Among those with three diagnoses, the most common combination was major depression, social phobia, and generalized anxiety disorder with 57 cases at phase 1 and 63 cases at phase 2 (*p* = 0.263). The frequency of subjects with more than one diagnosis increased significantly from phase 1 to phase 2 (*n* = 333, 21.5% *v*. *n* = 359, 23.2%, *p* < 0.001).

**Table 3.** Distribution of combinations of mental disorders between phase 1 and 2

	Diagnosis at T1										
	None (*n* = 864)	MD (*n* = 85)	SP (*n* = 77)	PTSD (*n* = 91)	GAD (*n* = 99)	MD + SP (*n* = 25)	MD + PTSD (*n* = 29)	MD + GAD (*n* = 130)	SP + PTSD (*n* = 4)	SP + GAD (*n* = 23)	PTSD + GAD (*n* = 5)
Diagnosis at T2:											
None	793	1			2	1	1				
MD	15	76						2			
SP	13		70					1			
PTSD	5			85			2				
GAD	24		1		90			2			
MD + SP			3			23				1	
MD + PTSD	1	1					23				
MD + GAD	5	5			4			116		1	
SP + PTSD	1								3		
SP + GAD	2		2		2				1	21	
PTSD + GAD	1			6	1						5
MD + SP + GAD	3	2				1	2	3			
MD + SP + PTSD			1					1			
MD + PTSD + GAD								4			
All	1							1			

MD, major depression; SP, social phobia; PTSD, post-traumatic stress disorder; GAD, generalized anxiety.

## Discussion

To our knowledge, this is the first longitudinal population study among IDPs of changes in prevalence and course of mental health disorders. The study revealed significant increases in the most common mental disorders over time that might be attributable to prolonged displacement and high levels of daily stress. While there is limited previous evidence to support a negative mental health development due to prolonged forced displacement, this is nevertheless indicated in several cross-sectional studies (Scholte *et al*., 2004, Roberts *et al*., 2008, Roberts *et al*., 2009). Lack of improvement in mental health might also be related to continuous poverty, lack of hope, high levels of unemployment and poor housing, further leading to increased risk of mental health disorders (Murali and Oyebode, 2004).

Our 1-year increase in mental disorders cannot be directly compared with UN's 10-year projection, but nevertheless concurs with the direction of the UN estimates. According to the global burden of disease forecast, the Sub-Saharan African regions will experience an increase in the burden of mental health and substance use disorders of around 13% from 2010 to 2020. The largest increase will come in the central part of Sub-Saharan Africa with major depression having the highest increase (Charlson *et al*., 2014).

There are few longitudinal mental health studies from Africa, but in a study from the western part of rural Kenya two repeated household surveys of common mental disorders were completed in the same area in 2004 and 2013 (Jenkins *et al*., 2015). The studies found a stable albeit much lower rate of mental disorders than in the current study of 10.8% in 2004 and 10.3% in 2013. The difference in prevalence compared to our study might be due to the use of different instruments as the Clinical interview schedule was used in the study from Kenya. The differences might also reflect real differences due to more hardship among the IDPs as compared to the general population studied in Kenya.

At the same time, the overall persistence of disease burden within 1 year, with few recovered cases, could be due to lack of mental health services in IDP settings that could provide an opportunity for recovery. However, the study also revealed that there is stability among those free from disease, although there were more new than recovered cases. While the study cannot explain the relative stability among the disease-free, this resilience should be pursued in future studies.

There is a need to provide mental health services among IDPs. The WHO Comprehensive Mental Health Action Plan 2013–2020 (WHO, 2013) proposes a systematic transition from mental hospitals to community-based services. To reach out there is a need to share tasks and interventions should be combined at different levels and include awareness of mental health stigma (WHO, 2018), and access to treatment (Patel, 2007).

### Strengths and limitations

An important strength of the study is its longitudinal design with a high response and follow-up rate and the use of validated diagnostic instruments and highly skilled psychologists during data collection. Thorough training of highly qualified research assistants prior to data collection is also a strength as well as the use of community volunteers. A limitation of the study is that only two IDP areas in Central Sudan were studied, making it difficult to generalize the findings to all IDPs despite the two study areas being randomly selected. However, the similarity of the findings in the two areas indicate that the results may also be valid for other disadvantaged settlements in other areas of Sudan. Another limitation was the loss of more than 300 respondents to follow up as those not being interviewed for the second time might have had higher rates of mental health problems. We also recognize that the MINI remains within a categorical diagnostic approach, whose distinctive clinical and descriptive paradigm has been increasingly questioned in favour of a more trans-diagnostic approach (Patel *et al*., 2018). That approach complicates the meaning of inter-diagnostic shifts detailed here, underscoring the importance of overall limited ‘recovery’ across diagnosis in this large population over time.

## Conclusion

The study revealed continued high levels of psychiatric disorders and increase over time that might be due to poverty, low education, social exclusion and unavailability of effectively implemented psychiatric services. Further research is necessary to reveal the mechanisms leading to continued high levels and how to reduce mental health problems among IDPs.

## Footnotes

† The notes appear after the main text.

1 The study included an intervention program, which involved doctors and medical assistants working at the primary health care level in order to provide psychiatric services.
